# Indoor Exposure and Adverse Birth Outcomes Related to Fetal Growth, Miscarriage and Prematurity—A Systematic Review

**DOI:** 10.3390/ijerph110605904

**Published:** 2014-06-03

**Authors:** Evridiki Patelarou, Frank J. Kelly

**Affiliations:** 1 Florence Nightingale School of Nursing and Midwifery, King’s College London, London SE18WA, UK; 2 NIHR Environmental Hazards Health Protection Research Unit, MRC-PHE Centre for Environment and Health, King’s College London, London SE19NH, UK; E-Mail: frank.kelly@kcl.ac.uk

**Keywords:** pregnancy, gestational age, environmental exposures, environmental tobacco smoke, air pollution, indoor air pollution, birth weight, small for gestational age, fetal growth, preterm delivery

## Abstract

The purpose of this review was to summarize existing epidemiological evidence of the association between quantitative estimates of indoor air pollution and all-day personal exposure with adverse birth outcomes including fetal growth, prematurity and miscarriage. We carried out a systematic literature search of MEDLINE and EMBASE databases with the aim of summarizing and evaluating the results of peer-reviewed epidemiological studies undertaken in “westernized” countries that have assessed indoor air pollution and all-day personal exposure with specific quantitative methods. This comprehensive literature search identified 16 independent studies which were deemed relevant for further review and two additional studies were added through searching the reference lists of all included studies. Two reviewers independently and critically appraised all eligible articles using the Critical Appraisal Skills Programme (CASP) tool. Of the 18 selected studies, 14 adopted a prospective cohort design, three were case-controls and one was a retrospective cohort study. In terms of pollutants of interest, seven studies assessed exposure to electro-magnetic fields, four studies assessed exposure to polycyclic aromatic hydrocarbons, four studies assessed PM_2.5_ exposure and three studies assessed benzene, phthalates and noise exposure respectively. Furthermore, 12 studies examined infant growth as the main birth outcome of interest, six examined spontaneous abortion and three studies assessed gestational age at birth and preterm delivery. This survey demonstrates that there is insufficient research on the possible association of indoor exposure and early life effects and that further research is needed.

## 1. Introduction

The root cause of many adverse birth outcomes is not well understood, although there is growing evidence that the environment can play an important role. The term “environment” is broad and may include nutrition, smoking and alcohol use, social networks, and air pollution both outdoor and indoor. The developing fetus is thought to be particularly susceptible to environmental pollutants and birth outcomes that may be influenced by exposure to environmental factors include gestational duration, infant growth, miscarriage/pregnancy loss and congenital anomalies [1]. A large body of evidence demonstrates that, in addition to parental smoking [2,3,4] and environmental tobacco smoke (ETS) [5], outdoor and indoor air pollutants may increase the risk of adverse birth outcomes, including low birth weight (LBW), premature births, and intrauterine growth retardation (IUGR) [1,6,7,8].

Several studies have associated maternal exposure to ambient air pollution (especially PM_2.5_) during pregnancy and a heightened risk of preterm delivery (PTD), low birth weight (LBW) and other adverse health effects [9]. In addition, a recent study with pooled data for 14 population-based mother-child cohort studies in 12 European countries confirmed previous findings that exposure to ambient air pollutants and traffic during pregnancy is associated with restricted fetal growth [10]. A number of studies conducted in developing countries have also addressed the effect of exposure to indoor air pollution (IAP) (mainly from solid fuel combustion processes) on diseases, such as respiratory infection, chronic obstructive pulmonary disease, cataract, asthma, heart diseases and adverse birth outcomes [11]. Pope *et al.* conducted meta-analyses to quantify the relation of indoor air pollution from solid fuel combustion processes with birth weight and stillbirth [12]. When women using solid fuel were compared with those using cleaner fuels it was found that solid fuel use was associated with increased risk of LBW and stillbirth (OR 1.38, 95% CI 1.25 to 1.52 and OR 1.51, 95% CI 1.23 to 1.85) [12]. A more recent review by Misra *et al.* aimed to establish a quantitative association between LBW attributable to IAP [11]. Seven studies were identified (six of them conducted in developing countries) and the meta-analysis indicated that the summary risk of LBW increased 1.45-fold due to IAP exposure [11].

Exposure to electromagnetic fields (EMF) in the indoor environment (home, work, social places) and its effect on pregnancy outcome still remains controversial and the majority of the published studies have focused on the potential effect of EMF on the risk of childhood leukemia [13,14,15]. These studies have showed relative risks slightly above 1.0 but most failed to assess personal exposure accurately instead using surrogates including wire code classification of the residence and retrospective spot measurements [14,16]. The effect of EMF on miscarriage/pregnancy loss has been studied only to a limited extent and examination has mostly been for exposure to video display terminals (VDT). In addition, the occurrence of adverse birth outcomes among women living or working in noisy environments has been also reported with some studies suggesting an association between noise exposure during pregnancy and low birth weight [17]. However, these studies had considerable limitations regarding exposure assessment as they did not manage to directly assess noise exposure and the possible periodic variation of noise exposure was not considered.

Given that most people, especially pregnant women and children, spend most of their time indoors, the consequences of indoor exposure to environmental hazards range from negligible to severe, and can even be fatal to the unborn child in certain circumstances. Considering the scale of the problem and the potential severity of the associated risk our aim was to assess the effect of exposure to indoor environmental hazards on adverse birth outcomes among studies conducted in “westernized” countries. In this systematic review, exposures were assessed through quantitative measures among studies conducted in “westernized” countries on the development of adverse birth outcomes including infant growth, gestational age and miscarriage.

## 2. Methods

### 2.1. Literature Search Strategy

A systematic review of the existing literature on indoor pollutants and adverse birth outcomes was carried out. We posed the following review question: “Given existing epidemiological evidence, what is the relationship between exposure of pregnant women to indoor pollutants and the risk of various adverse birth outcomes?” We drew up a review protocol in advance following standards outlined in the MOOSE Guidelines for Meta-Analyses and Systematic Reviews of Observational Studies [18]. Next we carried out a systematic, comprehensive bibliographic search using Medline (National Library of Medicine) database for the years 1946–March 2013, using the PubMed interface. Search terms used were chosen from the USNLM Institutes of Health list of Medical Subject Headings (MeSH) for 2013. These were: “Air Pollution, Indoor”; “Particulate Matter”; “Nicotine”; “Carbon Monoxide”; “Nitrogen Dioxide”; “Sulfur Dioxide”; “Polycyclic Hydrocarbons, Aromatic”; “Radon”; “Solvents”; “Asbestos”; “Ozone”; “Pesticides”; “Volatile Organic Compounds”; “Formaldehyde”; “Benzene”; “Toluene”; “Styrene”; “Dibutyl Phthalate”; “phthalate.mp.”; “Polyvinyl Chloride”; “Noise”; “Noise, Occupational”; “Electromagnetic Fields”; “Magnetic Fields”; “Pregnancy Outcome”; “Pre-Eclampsia”; “Pregnancy Outcome”; “Fetal Death”; “Premature Birth”; “Pregnancy Complications”; “Abortion, Spontaneous”; “Birth Weight”; “low birth weight.mp.”; “Infant, Low Birth Weight”; “Fetal Growth Retardation”; “Gestational Age”; “intrauterine growth.mp.”; “Embryonic and Fetal Development”; “Congenital Abnormalities”; “Hypertension, Pregnancy-Induced”; “Infant Mortality”; “Perinatal Mortality”; “Fetal Death”; “Infant, Premature”; and “preterm.mp.”. Full details of the search strategy and the key-words’ combination are provided in Table 1. The same search method was then repeated using the EMBASE database. Bibliographies of each retrieved study and reviews were also checked by hand for additional studies that met broad eligibility criteria.

**Table 1:** Search terms used to identify relevant studies for the review.

IAP ^§^ and Pregnancy Outcome
Exposure
1. * Air Pollution, Indoor/
2. * Particulate Matter/
3. * Nicotine/
4. * Carbon Monoxide/
5. * Nitrogen Dioxide/
6. * Sulfur Dioxide/
7. * Polycyclic Hydrocarbons, Aromatic/
8. * Radon/
9. * Solvents/
10. * Asbestos/
11. * Ozone/
12. * Pesticides/
13. * Volatile Organic Compounds/
14. * Formaldehyde/
15. * Benzene/
16. * Toluene/
17. * Styrene/
18. * Dibutyl Phthalate/or phthalate.mp.
19. * Polyvinyl Chloride/
20. * Noise/or * Noise, Occupational/
21. * Electromagnetic Fields/
22. * Magnetic Fields/
23. 1 or 2 or 3 or 4 or 5 or 6 or 7 or 8 or 9 or 10 or 11 or 12 or 13 or 14 or 15 or 16 or 17 or 18 or 19 or 20 or 21 or 22
Outcome
24. * Pregnancy Outcome
25. * Pre-Eclampsia/or * Pregnancy Outcome/or * Fetal Death/or * Premature Birth/or * Pregnancy Complications/or * Abortion, Spontaneous/
26. * Birth Weight
27. low birth weight.mp. or * Infant, Low Birth Weight/
28. Fetal Growth Retardation/or Gestational Age/or intrauterine growth.mp. or “Embryonic and Fetal Development”/
29. * Congenital Abnormalities/
30. * Hypertension, Pregnancy-Induced/
31. * Infant Mortality/or * Perinatal Mortality/or *Fetal Death
32. * Infant, Premature/or preterm.mp.
33. 24 OR 25 OR 26 OR 27 OR 28 OR 29 OR 30 OR 31 OR 32
Combined terms
34. 23 AND 33

Note: **§** Abbreviation: IAP, indoor air pollution.

### 2.2. Selection Criteria

From the identified papers, studies meeting the following eligibility criteria were selected:

- papers published in peer-reviewed journal
- papers published in English language
- human epidemiological studies of any study design
- studies conducted in developed countries (definition was based on the list of Developing Countries provided by the International Statistical Institute)

Studies not meeting these criteria were excluded and studies meeting the criteria were shortlisted for inclusion in the review. The list was further narrowed down on the basis of their exposure assessment methods. Specifically, only studies characterising exposure with quantitative methods during pregnancy were included. We also decided to include studies that assessed all day exposure with the use of personal monitors given the fact that people and especially pregnant women, usually spend almost all (90%) of their time indoors (home, work, social places) [19,20,21].

### 2.3. Literature Screening and Data Extraction

Studies were evaluated for inclusion by two independent reviewers for relevance to the subject. Study selection was accomplished through four levels of study screening. Disagreement was resolved by discussion. At level 1 screening, studies were excluded by reviewing the title of the article. At level 2 screening, abstracts of all studies accepted at level 1 were reviewed for relevance. For level 3 screening, the full text was obtained for relevant papers and any citations for which a decision could not be made from the abstract- level 2. For level 4 screening, a hand search of recent reviews or already retrieved original articles was performed and additional referenced, manuscripts were included in the systematic review. Data were then extracted systematically from each selected study using a pre-designed standard data collection form. Information on study design, methods, pollutants and outcome of interest, source and timing of exposure, location of study, results and confounding factors used during statistical analyses were obtained.

### 2.4. Study Evaluation and Critical Appraisal of the Evidence

Analyses of the data, as well as evaluation of the evidence presented in the articles, were performed with the use of the Critical Appraisal Skills Programme (CASP) in order to grade the evidence extracted [22,23]. The CASP tool uses a systematic approach to appraise three broad areas for consideration: study validity, an evaluation of methodological quality and presentation of results and an assessment of external validity [22,23]. There are 12 specific questions for cohort studies and 11 for case-control studies assessing the following: study validity, risk of bias in recruitment, exposure, outcome measurement, confounding factors, reporting of results and the transferability of findings. Each of the questions can be answered with “yes”, “no” or “can’t tell” and each study can have a maximum score of 12 (if cohort study) and 11 (if case-control study). In our review, two of the CASP questions were not included. The question, “Can the results be applied to the local population?” was not included because the focus of this review was not tied to a specific local population and the question, “Do the results of this study fit with other available evidence?” was not included for each individual study, as the purpose of this review was to compare results across studies. The articles were graded independently by the 2 reviewers who resolved any disagreements through consensus. The scores were used to grade the methodological quality of each study assessed (maximum score 10 for cohort and 9 for case-control studies). The grades given for each study rated the data related to this review article and may not reflect the overall quality of the study.

## 3. Results

### 3.1. Bibliographic Search

**Figure 1:**
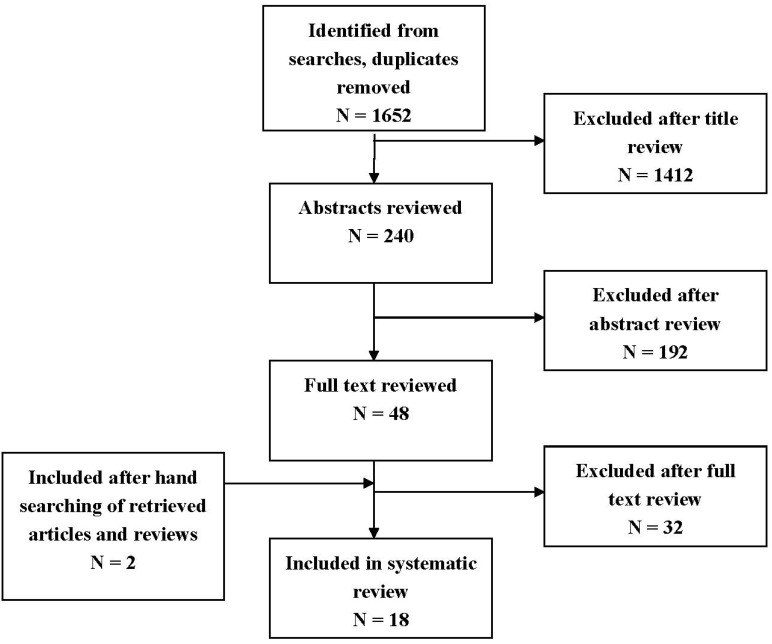
Flow chart for selection of studies.

Our combined search to MEDLINE and EMBASE retrieved 1,652 records. The initial screening of manuscript titles and abstracts excluded 1,604 records that did not meet the eligibility criteria. Common reasons for articles’ exclusion included studies conducted in developing countries, in non-English language and studies that did not develop a quantitative approach for assessing exposure. We excluded another 32 articles after examination of the full text. Additionally, two articles were retrieved by searching the reference lists of retrieved reviews and articles. Figure 1 shows the numbers of studies identified and selected/excluded in each phase of the search. Ultimately, eighteen articles were deemed suitable for inclusion in the review.

### 3.2. Overview of the Included Studies

Characteristics of the studies included in the analysis are given in Table 2 and Table 3. Among the relevant studies three were case-control studies [24,25,26], one was a retrospective cohort study [27] and fourteen were prospective cohort studies [17,28,29,30,31,32,33,34,35,36,37,38,39,40]. Eight articles used data of studies conducted in USA [26,27,28,30,36,37,38,40], five in Poland [31,32,33,34,35], two in Finland [24,25], one in Taiwan [17], one in France [39] and one combined data from two prospective studies conducted in USA and in Poland [29]. Overall, among the retrieved studies exposure characterisation varied widely, particularly in terms of the exposure assessment methodology. Exposure assessment methods used in the studies are also described in Table 2 and Table 3. In total, seven studies examined solely indoor exposure either at home [24,26,28] or at work [25,27] or at both places [36,37] and twelve studies referred to pregnancy cohort studies which assessed all day exposure to specific pollutants with the use of personal monitors [17,29,30,31,32,33,34,35,38,39,40]. In terms of the exposure of interest, seven studies investigated exposure to EMF [24,25,26,27,36,37], four articles exposure to fine particles smaller than 2.5 micrometers (PM_2.5_) [32,33,34,35], four polycyclic aromatic hydrocarbons (PAH) exposure [29,30,31,38], one to benzene exposure [39], one to phthalate exposure [40] and one to noise exposure [17]. Spontaneous abortion (SAB), pregnancy loss and miscarriage was the outcome of interest for five studies [24,25,27,36,37], infant growth was examined by eleven articles [17,28,29,30,31,32,33,34,35,38,39] and duration of gestation by three articles [29,30,40].

### 3.3. Effects of Quantified Exposure to Indoor Pollutants on Birth Outcomes

Seven studies were identified that addressed the potential effects of EMF or MF exposure and the risk of adverse birth outcome [24,25,26,27,28,36,37]. Below we present a short description of the methods and the main findings for each study (Table 4).

The first study that measured EMF exposure and adverse birth outcomes was performed in 1991 [27]. Authors conducted a case-control study and performed specific EMF measurements of telephone operators who used VDT with a cohort of operators who did not use VDT. The aim of the study was to examine the association between SAB and the measured EMF at VDT workstations. No excess risk of SAB among women who were exposed to EMF during the first trimester of pregnancy (OR 0.93, 95% CI 0.63 to 1.38) was found.

The following year, Lindbohm and colleagues conducted a study among women employed as bank clerks and clerical workers in three companies in Finland where specific measurement of the fields of the VDT were made [25]. The study showed that the OR for SAB for workers who had used a VDT with a high level of extremely low frequency MF (>0.9 μT) was 3.4 (95% CI 1.4–8.6) compared with workers using a terminal with a low level of MF (<0.4 μT) [25].

Furthermore, a case-control study performed by Juutinailen and colleagues investigated the association of residential exposure to EMF and early pregnancy loss [24]. Overall, this study provided some indication that exposure to high-intensity, residential, 50 Hz MF might be associated with increased risk of early pregnancy loss (for exposure levels ≥0.25, μT: OR 5.44, 95% CI 1.1 to 28) but the authors concluded that this associations should be interpreted cautiously due to the small study size and the limited number of highly exposed subjects [24].

Savitz and Ananth also examined the exposure to residential MF based on spot measurements and its association with adverse birth outcome [26]. These authors found that exposure to residential MF ≥0.2 μT was not more likely to end in miscarriage, LBW or PD. Similarly, a prospective cohort study among 2,967 births showed that exposure to EMF during pregnancy at work or at home assessed with the use of personal monitors, or home measurements showed no relation to the risk of LBW or FGR [28].

A more recent prospective cohort study examined the effect of MF on the risk of miscarriage [37]. For exposure assessment all participants were asked to wear a MF-measuring meter for 24 h and to keep a diary of their activities. Although no association between miscarriage risk and the average MF level was observed, miscarriage risk increased with an increasing level of maximum MF exposure with a threshold around 16 milligauss (mG). The rate ratio associated with MF exposure ≥16 mG (*vs.* <16 mG) was 1.8 (95% CI 1.2 to 2.7) and authors concluded that their findings provided strong prospective evidence that prenatal maximum MF exposure above a certain level (possibly 16 mG) may be associated with miscarriage risk.

The same year a case-control study was published which aimed to assess the relation between retrospective MF measures and clinical miscarriage [26]. Lee and colleagues performed area spot measures at work and/or home, and personal meter metrics including the average difference between consecutive levels, the maximum level and the time weighted average [36]. For area measures these authors found little association of exposure and miscarriage but for the personal metrics, positive associations were found. Specifically, exposures were divided into quartiles, with the lowest quartile used as the referent. Starting with the highest quartile adjusted OR and 95% CI were 3.1 (95% CI 1.6–6.0), 2.3 (95% CI 1.2–4.4), and 1.5 (95% CI 0.8–3.1) for the rate-of-change metric (0.94+ *vs.* 0.62–0.94 *vs.* 0.43–0.62 *vs.* <0.43mG) and the OR conveyed by being above a 24-h time-weighted average of 2 mG at home was 3.0 (95% CI 1.1–8.4).

### 3.4. Effects of Quantified All-day Exposure to Pollutants on Birth Outcomes

In total ten articles have been published which examine the potential effects of air pollution exposure assessed through all-day personal measurement on the risk of adverse birth outcome [29,30,31,32,33,34,35,38,39,40] (Table 5). However, only three studies were conducted and analysed to inform those ten articles. These were the Krakow study which enrolled non-smoking, pregnant women in Poland between 2000–2003, the NYC study (New York City) which recruited American or Dominican women who reside in New York City between 2000–2006 and the EDEN mother-child cohort that recruited French women <20 gestational weeks between 2003–2006. Women recruited in both Krakow and NYC study were given a backpack containing a portable personal exposure air monitor to be worn during the day and kept near the bed at night during a consecutive 48-h period. The EDEN participants were asked to carry a diffusive air sampler for seven consecutive days.

**Table 2:** Summary of studies’ characteristics and exposure- outcome assessment methodology of studies included in the review.

Reference	Study Characteristics	Exposure Assessment	Pollutants Studied (Units)	Outcome Assessment
[27]	USA 1987–1988: telephone interviews 1990: measurements of the electromagnetic fields Retrospective study design Women employed as directory—assistance operators and general telephone operators at two companies Sample population: 730	Occupational exposure status based on measurements conducted at some (8 of the 50) workstations. Measurements taken at operator’s abdomen.	MF (μT)	Self reported cross-checked with state records
[25]	Finland, 1975–1985 Case- control study Women employed as bank clerks and clerical workers in three companies Sample population: 585	Occupational exposure status based on laboratory measurements of the fields of 17 models of VDT. Measurements taken at 50 cm in front of the screen and at the site approximated for the fetus (25 cm down) at the same distance.	EMF (μT)	Self reported cross-checked with nationwide data records
[24]	Kuopio, Finland, 1988–1989 A nested case-control study 89 cases and 102 controls that had participated to the Work and Fertility study during the period 1984–1986 Sample population: 191	Magnetic field was measure in the residences where the women lived when participating in the Work and Fertility study. The magnetic field strength was measured at the front door of each residence in the living room, in the kitchen, and in the parents’ bedroom. The measurement in the bedroom was taken at the center of the bed whereas the measurements in the other rooms were taken near the center of the room, 1m above the floor. Measurements were also taken in other parts of the room to check that the field in the chosen measuring point represents the average level of the room.	MF (A/m)	Hospital records
[26]	Colorado, USA, 1976–1983 Case-control study 78 childhood cancer cases and 78 controls selected through birth certificates Sample population: 156	Electric and magnetic field measurements were sought at the time of the interviews at those residences. Measurements were taken near the front door, in the child’s bedroom, and in the parents’ bedroom. Any room reported in the questionnaire to have been occupied by the child an average of one or more hours per day was measured. In each selected room, measurements were taken as near as possible to the center of the room while avoiding close proximity to appliances or large metal objects.	EMF (mT)	Hospital records
[28]	Connecticut, USA, 1988–1991 Prospective study Women receiving their prenatal care at 11 private obstetrical practices and two health maintenance organizations Sample population: 2,967	Residential exposure status. EMF exposure using: personal monitors—women were asked to wear an average magnetic field exposure meter for the following 7 days leaving it at the bedside at night. Home measurement- An electric and magnetic digital exposure meter was placed in the center of a room for a 24-h period.	EMF (mG)	Hospital records and direct examination of the newborns
[17]	Taiwan, 1991 Prospective study Women in the first trimester of their pregnancy from obstetric clinics at 25 maternity hospitals Sample population: 200	Residential exposure status. Personal 24-h noise exposure was measured on work days at work and home.	Noise (dBALeq24)	Hospital records
[36]	California, USA, 1990–1991 Prospective sub-study Nested Case-Control Study Subjects recruited from a cohort of 3403 pregnant women who participated in a large prospective reproductive health study Sample population: 155 cases, 509 controls	Residential and occupational exposure status. EMF exposure using: Personal monitors—women were asked to use a meter for a 24-h period and record on an activity card the time when they entered a new environment. Women were leaving meters at the bedside at night. Home measurement- Spot measurements with the same meter were taken outside the front door and inside the home in the center of the kitchen, living room, and participant’s bedroom	EMF (mG)	Prospective reproductive health study records
[37]	San Francisco, USA, 1996–1998 Prospective cohort study All women with a positive pregnancy test at less 10 weeks of gestation and residing in the San Francisco area were contacted for participation in the study. Sample population: 969	All participants were also asked to wear a magnetic field measuring meter for 24 h and to keep a diary of their activities. Spot measurements were taken in the subject’s bed room, the kitchen and the most frequent occupied room that was neither a bedroom nor a kitchen. Measurements were made at the abdominal level in the center of each room as well as the location at the subject typically occupied. In addition, measurements were taken at the front entrance of the residence and at approximately 15-foot intervals proceeding clockwise around the residence.	MF (mG)	Health databases

Notes: EMF, electromagnetic fields; MF, magnetic fields.

**Table 3:** Summary of studies’ characteristics and exposure- outcome assessment methodology of studies included in the review.

Reference	Study Characteristics	Exposure Assessment	Pollutants Studied (Units)	Outcome Assessment
[38]	New York, USA NYC prospective study Non-smoking women aged 18–35, who registered at the obstetrics and gynecology clinics of two hospitals by the 20th week of pregnancy Sample population: 263	During the 3rd trimester of pregnancy women were asked to wear a small backpack containing a personal monitor during the day time hours for 2 consecutive days and to place the monitor near the bed at night.	8 carcinogenic PAHs (ng/m^3^): benz[a]anthracene, chrysene, benzo[b]fluroanthene, benzo[k]fluroanthene, B[a]P, indeno[1,2,3-cd]pyrene, disbenz[a,h]anthracene and benzo[g,h,i]perylene	Hospital records
[32]	Krakow, Poland, 2001–2003 Krakow prospective study The cohort consisted of 362 pregnant women who gave birth between 34 and 43 weeks of gestation Sample population: 362	Women were asked personal air monitoring over 48 h during the 2nd trimester of pregnancy	PM_2.5_ (ng/m^3^)	Hospital records
[33]	Krakow, Poland, 2001–2004 Krakow prospective study The cohort consisted of 493 pregnant women who gave birth between 37 and 43 weeks of gestation Sample population: 493	Women were asked personal air monitoring over 48 h during the 2nd trimester of pregnancy	PM_2.5_ (ng/m^3^)	Hospital records
[34]	Krakow, Poland, 2001–2004 Krakow prospective study The cohort consisted of 481 pregnant women who gave birth between 37 and 43 weeks of gestation Sample population: 481	Women were asked personal air monitoring over 48 h during the 2nd trimester of pregnancy In the monitoring period women have spent on average 3 h outdoors and 2 h in the public transportation and those who used the public transportation had insignificantly higher level of PM_2.5_ exposures.	PM_2.5_ (ng/m^3^)	Hospital records
[35]	Krakow, Poland, 2001–2004 Krakow prospective study The cohort consisted of 431 pregnant women who gave birth between 37 and 43 weeks of gestation Sample population: 431	Women were asked personal air monitoring over 48 h during the 2nd trimester of pregnancy	PM_2.5_ (ng/m^3^)	Hospital records
[29]	Krakow, Poland 2000–2003 New York, USA 2004 Data from two prospective cohort studies (Krakow and NYC) that enrolled non-smoking, healthy, and non-occupationally exposed women and their newborns. Sample population: 720	Women were asked to wear a small backpack containing a personal monitor during the day time hours for 2 consecutive days and to place the monitor near the bed at night.	Levels of pyrene and 8 carcinogenic PAHs (ng/m^3^): benz[a]anthracene, chrysene, benzo[b]fluroanthene, benzo[k]fluroanthene, B[a]P, indeno[1,2,3-cd]pyrene, disbenz[a,h]anthracene and benzo[g,h,i]perylene	Hospital records
[30]	New York, USA, 2004 NYC prospective study Women non-smoking, healthy, and non-occupationally exposed women and their newborns. Sample population: 616	During the 3rd trimester of pregnancy women were asked to wear a small backpack containing a personal monitor during the day time hours for 2 consecutive days and to place the monitor near the bed at night.	Levels of pyrene and 8 carcinogenic PAHs (ng/m^3^): benz[a]anthracene, chrysene, benzo[b]fluroanthene, benzo[k]fluroanthene, B[a]P, indeno[1,2,3-cd]pyrene, disbenz[a,h]anthracene and benzo[g,h,i]perylene	Hospital records
[31]	Krakow, Poland, 2000–2003 Krakow prospective study Causacian pregnant women of ethnic Polish background aged 18–34 during the 8th to 13th weeks of gestation were included in the study. Sample population: 344	Women were simultaneously monitored for their personal (*n* = 344), home indoor (*n* = 76) and outdoor (*n* = 70) levels of PAHs and PM_2.5_ during the 2nd trimester of pregnancy. The subset of women that were personal monitored were asked to wear a small backpack containing a personal monitor during the day time hours for 2 consecutive days and personal monitoring was repeatedly taken once during each trimester.	Levels of pyrene and 8 carcinogenic PAHs (ng/m^3^): benz[a]anthracene, chrysene, benzo[b]fluroanthene, benzo[k]fluroanthene, B[a]P, indeno[1,2,3-cd]pyrene, disbenz[a,h]anthracene and benzo[g,h,i]perylene	Hospital records
[39]	France, 2005–2006 EDEN prospective cohort study Women at <20 gestational weeks were recruited from two maternity hospitals between 2003–2006. Sample population: 271	Women were asked to carry a diffusive air sampler for 7 consecutive days and to keep it close to their bed when they slept.	Benzene (ng/m^3^)	Hospital records, measurements at birth, ultrasound examinations
[40]	New York, US 2000–2006 NYC prospective study Women 18–35 years of age who self identified as either African American or Dominican and who had resided in northern Manhattan or the South Bronx for ≥1 year before pregnancy. Sample population: 331	Women were asked to wear a small backpack containing a personal monitor during the day time hours for 2 consecutive days and to place the monitor near the bed at night.	Di(2-ethylhexyl)Phthalate (ng/m^3^)	Hospital records

**Table 4:** Summary of published measures of effect and critical appraisal grade for studies that assessed indoor exposure.

References	Time of Exposure	Outcome	Main Results OR (95% CI)	Covariates	CASPgrade
[27]	1st trimester	SAB (*n* = 136)	non users *vs.* 0.07 μT *vs.* 0.08 μT	None adjustment	4/9
			OR 1.00 *vs.* 0.92 95% CI (0.58–1.47) *vs.* 0.98 95% CI (0.58–1.64)		
[25]	1st trimester	SAB (*n* = 91)	<0.4 μT *vs.* 0.4–0.9 μT *vs.* >0.9 μT	Use of video display terminals, hours of use per week, quantity of work, frequency of technical breakdowns in automatic data processing devices, exposure to organic solvents, number of previous births, previous spontaneous abortions, use of an intrauterine device	5/9
			OR 1.0 *vs.* 1.9 95% CI (0.9–3.9) *vs.* 3.4 95% CI (1.4–8.6)		
[24]	Not specific trimester exposure	Pregnancy loss (*n* = 89)	Magnetic field exposure (A/m)	Smoking	6/9
			Front door value < 0.2 *vs.* ≥ 0.2		
			OR 1.11 95% CI (0.6 to 2.3)		
			Average < 0.2 *vs.* ≥ 0.2		
			OR 5.44 95% CI (1.1 to 28)		
[26]	Not specific trimester exposure		Measured magnetic fields- Spot measurements	None	5/9
			≥2 mT *vs.* <2 mT		
			Miscarriage OR 0.8 95% CI (0.3 to 2.3)		
			Low birth weight OR 0.3 95% CI (0.0 to 2.4)		
			Preterm delivery OR 0.7 95% CI (0.1 to 4.0)		
[28]	At conception, at ≤16 weeks, or 3rd trimester	LBW IUGR LBW IUGR	24-h home EMDEX monitor (mG)	Maternal religion, race, height, weight, gravity, age, work in pregnancy, third trimester smoking, caffeine consumption	8/10
			<1.0 *vs.* 1.0–1.9 *vs.* ≥2.0		
			Referent *vs.* 0.94 (0.24–3.61) *vs.* not estimable		
			Referent *vs.* 0.79 (0.32–1.92) *vs.* 0.62 (0.16–2.31)		
			7-day personal AMEX monitor (mG)		
			<1.0 *vs.* 1.0–1.9 *vs.* ≥2.0		
			Referent *vs.* 0.65 (0.15–2.88) *vs.* 1.35 (0.30–6.11)		
			Referent *vs.* 0.40 (0.14–1.15) *vs.* 1.16 (0.43–3.11)		
[17]	1st, 2nd, 3rd trimester	Birth weight (grams)	No risk reported	None adjustment	4/9
[36]	30th week of pregnancy	SAB (*n* = 155)	Measured fields 24 h	Maternal age, gestation at interview, coffee consumption around conception, income, race, and each of the other personal 24-h exposures (excluding total 24-h)	6/9
			≥2 mG *vs.* <2 mG		
			Home: OR 0.8 95% CI (0.2–3.5)		
			Work: OR 0.7 95% CI (0.3–1.5)		
			Total: 24-h OR 1.0 95% CI (0.5–2.1)		
			Front door spot: OR 1.2 95% CI (0.6–2.5)		
			Inside spots: OR 1.1 95% CI (0.5–2.2)		
			Time-weighted average		
			<0.72 *vs.* 0.72–0.93 *vs.* 0.93–1.28 *vs.* >1.28		
			OR 1.0 *vs.* OR 1.7 95% CI (0.9–3.3) *vs.* OR 1.7 95% CI (0.9–3.3) *vs.* OR 1.7 95% CI (0.9–3.2)		
			Maximum value (mG)		
			<14.31 *vs.* 14.31–23.42 *vs.* 23.42–35.05 *vs.* >35.05		
			OR 1.0 *vs.* OR 1.4 95% CI (0.7–2.8) *vs.* OR 1.9 95% CI (1.0–3.5) *vs.* OR 2.3 95% CI (1.2–4.4)		
[37]	1st trimester	SAB (*n* = 159)	Measured fields 24 h	Previous miscarriage, education, maternal age, gravidity, race, smoking since last menstrual period	6/9
			<16 mG *vs.* ≥16 mG		
			RR 1.8 95% CI (1.2–2.7)		
			Total sum of exposure over 16 mG in tertiles		
			160–1,079 *vs.* 1,080–4,759 *vs.* ≥ 4,760 mG		
			RR 1.7 95% CI (1.1–2.8) *vs*. RR 1.8 95% CI (1.1–2.9) *vs.* RR 2.0 95% CI (1.2–3.17)		

Notes: Abbreviations: SAB: spontaneous abortion, LBW: low birth weight, IUGR: intrauterine growth restricted.

**Table 5:** Summary of published measures of effect and critical appraisal grade for studies that assessed all-day personal exposure.

References		Time of Exposure		Outcome		Main Results OR (95%CI)			Covariates	CASPgrade
[38]		3rd trimester		Birth weight (grams), length (cm), head circumference (cm)		**All**			BMI, parity, cotinine, sex of baby, gestational age	6/10
						Birth weight: Beta-coefficient −0.05, *p*-value = 0.08				
						Birth length: Beta-coefficient −0.02, *p*-value = 0.08				
						Birth head circumference: Beta-coefficient −0.01, *p*-value = 0.12				
						**African-American**				
						Birth weight: Beta-coefficient −0.10, *p*-value = 0.02				
						Birth length: Beta-coefficient −0.02, *p*-value = 0.24				
						Birth head circumference: Beta-coefficient −0.02, *p*-value = 0.06				
						**Dominican**				
						Birth weight: Beta-coefficient −0.009, *p*-value = 0.81				
						Birth length: Beta-coefficient −0.02, *p*-value = 0.11				
						Birth head circumference: Beta-coefficient 0.003, *p*-value = 0.80				
[32]		2nd trimester		Birth weight (grams), length (cm), head circumference (cm)		**LogPM_2.5_**		Number of pregnancies, height, and prepregnancy weight of mother, sex of newborn, gestational age	7/10
						Birth weight: Beta-coefficient −200.821 95% CI (−385.968 to −15.674)			
						Birth length: Beta-coefficient −1.439 95% CI (−2.583 to −0.294)			
						Birth head circumference: Beta-coefficient −0.729 95% CI (−1.347 to −0.112)			
[33]		2nd trimester		Birth weight (grams), length (cm), head circumference (cm)		**PM**_2.5_		Maternal education, parity, maternal height, pre- pregnancy weight, weight gain over pregnancy, gestational age, gender of child, season of birth	7/10
						**27.0–46.19 μg/m^3^*vs.* ≥46.2 μg/m^3^**			
						Birth weight: Beta-coefficient −16.51 95% CI (−94.64 to 61.61) *vs.* −109.956 95% CI (196.649 to −23.263)			
						Birth length: Beta-coefficient −0.288 95% CI (−0.790 to 0.214) *vs.* −0.810 95% CI (−1.367 to −0.253)			
[34]		2nd trimester		Birth weight (grams), length (cm), head circumference (cm)		**LogPM**_2.5_		Maternal education, gestational age, parity, maternal height, prepregnancy weight, sex of infant, prenatal environmental tobacco smoke, season of birth	7/10
						Birth weight: Beta-coefficient −155.9 95% CI (−307.2 to −4.7)			
						Birth length: Beta-coefficient −1.24 95% CI (−2.19 to −0.28)			
						Birth head circumference: Beta- coefficient −0.53 95% CI (−1.04 to −0.02)			
[35]		2nd trimester		Systolic blood pressure (mmHg)Diastolic blood pressure (mmHg)		**LogPM**_2.5_		Maternal age, education, parity, gestational weight gain, prepregnancy BMI, environmental tobacco smoke, blood lead level	7/10
						Systolic blood pressure: Beta-coefficient −6.126 95% CI (0.610 to 11.642)			
						Diastolic blood pressure: Beta-coefficient 4.083 95% CI (−0.019 to 8.185)			
[35]		2nd trimester		Systolic blood pressure (mmHg)Diastolic blood pressure (mmHg)		**NYC African-American**				
						(ln) Birth weight: Beta-coefficient −0.055, *p*-value = 0.004				
						(ln) Birth length: Beta-coefficient −0.011, *p*-value = 0.112				
						(ln) Birth head circumference: Beta-coefficient −0.010, *p*-value = 0.125				
						**NYC Dominican**				
						(ln) Birth weight: Beta-coefficient 0.018, *p*-value = 0.094				
						(ln) Birth length: Beta-coefficient 0.003, *p*-value = 0.712				
						(ln) Birth head circumference: Beta-coefficient 0.004, *p*-value = 0.168				
[29]		Krakow: 8th to 24th week of pregnancyNYC: 3rd trimester		Gestational age (weeks), birth weight (g), length (cm) and head circumference (cm)		**(ln)Σ8c-PAHs**			Not specified	7/10
						**Krakow Caucasian**				
						(ln) Birth weight: Beta-coefficient −0.02, *p*-value = 0.007				
						(ln) Birth length: Beta-coefficient −0.009, *p*-value = 0.003				
						(ln) Birth head circumference: Beta-coefficient −0.006, *p*-value = 0.010				
						**NYC African-American**				
						(ln) Birth weight: Beta-coefficient −0.055, *p*-value = 0.004				
						(ln) Birth length: Beta-coefficient −0.011, *p*-value = 0.112				
						(ln) Birth head circumference: Beta-coefficient −0.010, *p*-value = 0.125				
						**NYC Dominican**				
						(ln) Birth weight: Beta-coefficient 0.018, *p*-value = 0.094				
						(ln) Birth length: Beta-coefficient 0.003, *p*-value = 0.712				
						(ln) Birth head circumference: Beta-coefficient 0.004, *p*-value = 0.168				
[30]		3rd trimester		Gestational age (weeks), preterm delivery (*n* = 20), small for gestational age (*n* = 53)		**(ln)Σ8c-PAHs **		* Maternal pre-pregnancy BMI, months of gestational ETS exposure, parity, winter delivery ** BMI, gestational weight gain, months of gestational ETS exposure, parity		7/10
						**African- American**				
						Gestational age *: Beta- coefficient −0.354 95% CI (−0.714 to 0.006)				
						Preterm delivery *: OR 4.676, 95% CI (1.839 to 11.886)				
						Small for gestational age **: OR 1.94, 95% CI (1.09– 3.47)				
						**Dominican**				
						Gestational age *: Beta-coefficient −0.006 95% CI (−0.190 to 0.178)				
						Preterm delivery *: OR 0.523, 95% CI (0.182 to 1.504)				
						Small for gestational age **: OR 0.82, 95% CI (0.44–1.51)				
[31]		2nd trimester		Birth weight (g) Birth length (cm) Birth head circumference (cm)		**Natural log- PAH exposure correlated with the reduction from the mean outcome**		Gestational age, gender, parity, maternal pre- pregnancy BMI, and c-section delivery included only for the head circumference		7/10
						Birth weight: Beta-coefficient −67 95% CI (−110 to −23)				
						Birth length: Beta-coefficient −0.48 95% CI (−0.76 to −0.20)				
						Birth head circumference −0.20 95% CI (−0.34 to −0.05)				
						Fetal growth ratio: −1.85 95% CI (−3.09 to −0.60)				
						Ponderal index: 0.01 95% CI (−0.01 to 0.04)				
						Cephalisation index: 1 95% CI (−2 to 4)				
[39]		27th week of pregnancy		Birth weight (g), head circumference (mm) and ultrasound examinations of head circumference (mm) and biparietal diameter (mm) between the 29th and 36th gestational weeks and before the 15th gestational week		**Benzene exposure (μg/m^3^)**		Gestational age at the examination, sex, maternal passive smoking, urinary cotinine levels, prepregnancy weightm height, parity, maternal occupational exposure to paints or pesticides, month of conception, maternal education, center		8/10
						**<1.4 *vs.* 1.4–2.59 *vs.* ≥2.6**				
						**Birth weight:** Beta-coefficient 95%CI				
						0 *vs.* −74 (−197 to 50) *vs.* −90 (−215 to 36) *vs.* −68 (−135 to −1)				
						**Head circumference at birth:** Beta-coefficient 95%CI				
						0 *vs.* −0.9 (−4.5 to 2.7) *vs.* −3.7 (−7.3 to 0.0) *vs.* −1.9 (−3.8 to 0.0)				
						**Head circumference the 2nd trimester ultrasound scan:** Beta-coefficient 95%CI				
						0 *vs.* −1.3 (−4.2 to 1.6) *vs.* −2.5 (−5.4 to 0.5) *vs.* −1.5 (−3.1 to 0.0)				
						**Biparietal diameter the 2nd trimester ultrasound scan:** Beta-coefficient 95%CI				
						0 *vs.* −0.5 (−1.5 to 0.5) *vs.* −1.0 (−2.0 to 0.0) *vs.* −0.6 (−1.1 to −0.1)				
[39]		27th week of pregnancy		Birth weight (g), head circumference (mm) and ultrasound examinations of head circumference (mm) and biparietal diameter (mm) between the 29th and 36th gestational weeks and before the 15th gestational week		**Head circumference at the 3rd trimester ultrasound scan:** Beta-coefficient 95%CI				
						0 *vs.* −1.6 (−5.4 to 2.3) *vs.* −4.8 (−8.8 to −0.8) *vs.* −1.9 (−4.0 to 0.3)				
						**Biparietal diameter at the 3rd trimester ultrasound scan:** Beta-coefficient 95%CI				
						0 *vs.* −0.2 (−1.5 to 1.0) *vs.* −1.3 (−2.6 to −0.1) *vs.* −0.6 (−1.2 to 0.1)				
						**ln(benzene)**				
						**Birth weight:** Beta-coefficient 95% CI −68 (−135 to −1)				
						**Head circumference at birth:** Beta-coefficient 95% CI −1.9 (−3.8 to 0.0)				
[40]		3rd trimester		Length of gestation (weeks)		**Log DEHP exposure (per unit increase)**		Maternal ethnicity, maternal age, maternal prepregnancy weight and height, active smoking during pregnancy, prenatal asthma, diabetes, hypertension, planned caesarean section, premature rupture membrane		7/10
						Gestational age: Beta-coefficient −0.15 95% CI (−0.39 to 0.09)				

Note: *and **, they refered to the covariates for which they were adjusted to.

The NYC study has informed four different publications relevant to the present review. The first publication evaluated the effects of prenatal exposure to airborne PAHs monitored during pregnancy, along with ETS estimated by plasma cotinine, and an organophosphate pesticide estimated by plasma chlorpyrifos on birth outcomes [38]. This study showed that among African Americans, high prenatal exposure to PAHs was associated with lower birth weight (*p* = 0.003) and smaller head circumference (*p* = 0.01) after adjusting for potential confounders.

The second publication consists of a further analysis of the NYC study population which aimed to examine whether prenatal exposure to air pollution in general, and PAHs in particular, increased the risk of IUGR, including SGA, and PTD [30]. Choi and colleagues concluded that a 1 natural-log (ln)-unit increase in prenatal PAH exposure was associated with a 2-fold increase in risk of symmetric IUGR among full-term African Americans (*p* < 0.05) [30]. In addition, they found that PTD risk was 5-fold greater among African Americans per ln-unit increase in prenatal PAH exposure and the same unit increase in exposure significantly increased the ratio of head circumference to birth weight by 0.04% in African Americans [30]. A more recent analysis of the NYC data confirmed that PAH exposure exerts the greatest adverse effect on fetal growth during the first trimester [31]. Furthermore, Whyatt *et al.* aimed to assess the relationship between di(2-ethylhexyl)phthalate exposure during pregnancy and gestational age at delivery and suggested that prenatal phthalate exposure was associated with a shorter period of gestation [30].

The Krakow study has informed 6 different articles relevant to our review [29,31,32,33,34,35]. The first publication in 2004 estimated exposure of pregnant women to PM_2.5_ and assessed this effect on birth outcomes. A significant inverse correlation between prenatal exposure to fine particles and fetal growth, as reflected in significantly lower mean weight and length at birth was reported [32]. Further analysis of these data was carried out and authors found that while the negative effect of higher prenatal PM_2.5_ exposure (above the 3rd tertile) on birth weight was significant in women with lower vitamin A intakes (beta = −185.07, *p*-value < 0.001) the effect became insignificant in the women with higher intakes [33]. The authors also concluded that the findings were similar for length at birth [33]. Further, analysis of the same data verified previous published evidence of the effect of PM_2.5_ exposure on the birth outcomes and showed that observed deficits in birth outcomes were attributable to prenatal PM_2.5_ exposure and not to ETS [24]. A further publication in 2009 evaluated whether gestational exposure to PM_2.5_ has a prohypertensive effect [35] with the authors suggesting that exposure to PM_2.5_ in the 2nd trimester of pregnancy has an effect on blood pressure values monitored in the 3rd trimester. There was a slightly stronger relationship between PM_2.5_ and systolic blood pressure than diastolic blood pressure, and it appeared that women with the excessive gestational weight gain were more susceptible to prohypertensive action of PM.

In a further series of analyses the data from the Krakow and NYC studies were combined and used to examine the association of prenatal exposure to airborne PAHs and infant size at birth [29]. Results confirmed the adverse reproductive effect of relatively low PAH concentrations and showed that prenatal PAH exposure was associated with significantly reduced birth weight in both Krakow Caucasians (*p* < 0.01) and in NYC African Americans (*p* < 0.01) but not in NYC Dominicans. Within the lower exposure range common to the two cities (1.80–36.47 ng/m^3^), the effect per unit PAH exposure on birth weight was 6-fold greater for NYC African Americans than for Krakow Caucasians (*p* = 0.01).

Finally, the EDEN cohort aimed to assess the relation between maternal personal exposure to airborne benzene during pregnancy and fetal growth. The study team found that log-transformed benzene exposure was associated with a gestational age–adjusted decrease of 68 g in mean birth weight (95% CI −135 to −1 g) and of 1.9 mm in mean head circumference at birth (95% CI, −3.8 to 0.0 mm) [39].

Further to the above, only one study has examined the effect of noise exposure in indoor environment [17]. This study examined the effect of noise exposure during pregnancy on infant birth weight in a cohort of 200 pregnant women in the first trimester of their pregnancy. Individual 24-h noise exposure of all women was prospectively measured, but no statistically significant correlation between personal noise exposure measured in decibels during pregnancy (less than 85 dBALeq) and birth weight was found.

In terms of critical appraisal grades the majority of the studies did not manage to achieve a high grade and this was due to their methodological pitfalls including exposure and outcome assessment methodology and the lack of confounding factors’ control over study design and analysis. Among studies that assessed indoor exposures Bracken *et al.* was given a grade of 8/10 [28] and among studies that assessed all day exposure through personal monitoring and Slama *et al.* was given a grade of 9/10 [39]. Both studies were prospective cohort studies, adopted an appropriate exposure assessment methodology (even if not ideal) and combined hospital records with infant’s measurements for outcome assessment purposes. A summary of the exposure categories, associated measures of effect for each health outcome and CASP grades of all included studies is presented in Table 4 and Table 5.

## 4. Discussion

In this review we have brought together the existing body of evidence of the effect of quantified indoor exposures and adverse birth outcomes. Our findings highlight the limited number of studies to date that attempt to quantify indoor exposure and/or all-day personal exposure to specific pollutants during pregnancy. The main pollutants of interest among the robust studies identified for consideration were exposure to EMF, fine particles (PM_2.5_), phthalates, PAHs and noise. These studies show an increased risk of exposure to EMF with SAB/early pregnancy loss/miscarriage but these findings should be interpreted with caution as a number of methodological limitations exist across these studies [24,25,36,37]. Noise exposure was assessed in only one study and no adverse effect of exposure (lower than 85 dBALeq_24_) were linked with birth weight. In contrast, a number of environmental exposures were identified to have links with effects on the unborn child. Three publications found an increased risk of exposure to PAHs and restricted infant growth [29,30,31,38], three studies documented a significant inverse association between prenatal exposure to fine particles and several measures of fetal growth [32,33,34] and one study found an association between benzene exposure and reduced fetal growth [39]. A further one study observed an inverse association between all day exposure to phthalate exposure and shorter gestational age [40] and one study correlated personal daily exposure to PM_2.5_ in the 2nd trimester of pregnancy with gestational hypertension in the 3rd trimester of pregnancy [35].

Most previous studies on the effect of EMF’s exposure and adverse health outcomes were case-control studies in design and exposure was often measured retrospectively or indirect measurements of EMF level were employed such as wire code configuration. Although more recent studies have attempted direct measurements, only residential spot measurements were obtained to represent a participant’s overall personal EMF exposure level. It is well known that residential spot measurements do not necessarily capture residential exposure and overall personal exposure from different sources. All of these may compromise EMF measurements and could lead to misclassification of the EMF exposure level that would tend to mask an underlying effect. The association between EMF exposure and the risk of miscarriage has been studied only to a limited extend and the examination has mostly been for exposure to VDT. However, as VDT only emits a limited amount of EMF it is unlikely to be a major source of MF in a woman’s daily life. Therefore, it would be difficult to detect an association of miscarriage with VDT use. The first study to evaluate personal EMF exposures for three different a priori summary metrics and for different types of daily environments (at home, at work and outside work and home environment) indicated that exposure to a VDT with a high MF level (>9 mG) during pregnancy, had a more than 3-fold increased risk of miscarriage and that time-weighted average MF exposure above 2 mG conveyed an excess risk [36]. However, as this study obtained MF measurements months after the occurrence of the miscarriage they may not be an accurate representation of earlier exposures during pregnancy. In addition, a prospective study that measured EMF exposure more close to the relevant time showed an increased risk of miscarriage associated with an MF exposure level ≥16 mG [37]. Consequently, despite the lack of a clear understanding of the underlying mechanism(s) there is evidence of a possible effect of MF on early fetal loss.

The effect of noise exposure during pregnancy on birth weight has been examined to a very limited extent and findings are inconclusive. The proposed underlying mechanism of how noise effects occur during pregnancy involves decreased uteroplacental blood flow resulting in fetal hypoxia and increased secretion of maternal catecholamine which further increases blood pressure and decreases placental function. As previously presented only one prospective study assessed individual 24-h noise exposure and found no evidence for an effect on infant’s birth weight [17]. One major limitation of this study was that noise measurement represented a summary noise index experienced by pregnant women and no consideration was given to levels of individual noise stimulation. Furthermore impulse noise, which was not considered, might result in an underestimated measurement of noise exposure. Consequently, as the association of noise exposure to fetal development still remains unclear further studies that take into account the contribution of extrinsic factors (frequency, intensity, duration of noise stimulus) and intrinsic factors (individual differences in physiological tolerance to noise) of study subjects should be considered.

An important finding of this review is the agreement of a significant inverse correlation between prenatal exposure to fine particles (PM_2.5_) and fetal growth as reflected in significantly lower mean weight and length at birth [32,33,34,35]. It is well known that air pollutants can lead to DNA damage and that a correlation between PAH-DNA adducts and fetal growth exists [38], however, the biologic mechanisms whereby PM_2.5_ might cause adverse birth outcomes remain to be fully elucidated. Several biological mechanisms that have been proposed through which air pollution could influence pregnancy, including the induction of systemic inflammation, oxidative stress, coagulation, altered endothelial function and hemodynamic responses [41]. All of which may eventually result in suboptimal placentation and increased maternal susceptibility to infections [42]. Overall however, the suggested link between fine particle exposure and adverse birth outcomes should be interpreted cautiously as exposure assessment was performed over a short period of 48 h during the 2nd trimester of pregnancy and as a result important information regarding the levels of exposure and how they contribute to the overall pregnancy exposure is missing from the wider literature.

PAHs are multiphasic fused aromatic rings of carbon compounds generated by a variety of combustion sources [43]. Several studies have shown that both active and passive tobacco smoke are important sources of exposure to PAHs, however, diet is the main source of human exposure to PAHs in the non-occupationally exposed populations [44]. Foods can be contaminated by PAHs present in polluted air, soil or water and PAHs can also be formed in foods as a consequence of processing and cooking methods such as drying, smoking, grilling, roasting or frying [44]. Prenatal exposure to PAHs quantified by personal air monitoring, significantly predicted dose-response elevations in cord blood chromosomal aberrations [45]. The mechanism of fetal toxicity to PAHs is not clear and may involve the induction of apopotosis after DNA damage, the antiestrogenic effects of PAHs, the binding to the human Ah receptor to induce P450 enzymes or to receptors for placental growth factors, resulting in decreased exchange of oxygen and nutrients [7]. The first study that reported positive findings of high prenatal exposure to PAHs and LBW had the advantage of being based on individual personal exposure data from personal monitoring over a 48 h period during 3rd trimester and biomarkers measured at a single point [38]. Choi *et al.* showed also the increased risk of exposure to PAHs and SGA and PTD but also addressed some of the limitations of previous studies including exposure misclassification due to retrospective or cross- sectional exposure assessment [29,30]. Thus there is evidence of an association between PAH exposure during the first trimester and FGR reduction and cephalization index elevation, however the identification of a “window of critical vulnerability” to PAHs remains a challenging question [31]. The reported effect of PM_2.5_ exposure on maternal hypertension should also be interpreted with caution due to the relatively small study sample and the fact that blood pressure measurements had been performed by a number of different medical personnel and therefore some bias might have occurred [35].

This review also presented the inverse relationship between exposure to benzene and infant growth [39] and future studies are needed to confirm or refute these findings as benzene is present both in outdoor (traffic, industrial emissions) and indoor environment (ETS, residential heating, emissions from consumer products) and consists of a proxy of pollutants in occupational and non-occupational settings. Interestingly, benzene levels much higher than common outdoor levels have been reported in car cabins [46] and findings from an European study estimated that exposures in transit contribute to 29% of total personal benzene exposure [47]. Furthermore, benzene exposure has been shown to be higher for women who use a car [29] and in homes with a garage with a connecting door to the living rooms [48].

In summary, previous studies have used a variety of methodologies to assess indoor exposure to environmental contaminants including EMF, noise, PM_2.5_, PAHs, benzene and phthalates but all of this research has a number of methodological limitations regarding outcome-exposure assessment and confounding factors analysis and control. More specific, the majority of previous studies failed to assess accurately whole pregnancy exposures and to capture spatial and temporal variations in exposure as no repeated measurements during pregnancy were performed. Furthermore, the majority of previous studies did not manage to address the impact of indoor pollution exposure on fetal growth using ultrasound measurements during pregnancy as direct and accurate estimates of growth. Some of the included studies performed all day personal exposure with the use of personal monitors from 24 h to 7 days but did not manage to distinguish the clear effect of solely indoors exposure and to capture all pregnancy trimester exposure. Indeed, exposure assessment was performed selectively for maximum 7 days either 2nd or 3rd trimester of pregnancy and outcome assessment was mainly based on hospital records. However, the majority of these studies identified possible associations of exposure with certain pregnancy outcomes and after considering the scale of the problem and the potential severity of the associated risks the need for good characterization and accurate quantification of indoor air pollutants is more needed than ever.

Further studies are required to fully understand and quantify the magnitude of individual exposure to air pollution in different types of indoors microenvironments. Future work should take into account different sources of indoor pollution, different occupancy and lifestyle scenarios and mobility of the women during pregnancy. Such an approach will prevent any potential misclassification that could arise when exposure is based solely on the home address at time of delivery [42]. The effect of exposure to outdoor sources of pollutant and specific types of outdoor pollutants (such as those experienced during commuting) should be taken into account when looking for the effect of indoor exposures on pregnancy outcome. It is well known that exposure to air pollution during commuting can be an important contribution to the total air pollution exposure, because most commuting takes place during rush hours when pollution concentrations can reach high levels and subjects are close to traffic emissions [49,50]. In addition, in order to provide insight into the specific effects of maternal indoor air pollution exposure and to identify critical windows of exposure, it is of interest to assess fetal growth in different periods of pregnancy rather than only at birth with the use of direct methods of assessment such as ultrasound measurement. Therefore, there is a need to carry out large, well-designed epidemiological studies which both taken into account relevant confounders and characterisation of exposure, and take care to use precise pregnancy outcomes. For this reason, the adaptation of innovative techniques for exposure assessment that combine direct all-day personal exposure measurements, direct measurements of micro-environmental concentrations and personal activity information is emerging [51,52].

## 5. Conclusions

While the number of studies may be insufficient to provide a definitive conclusion, this review provides a useful summary of existing quantitative research findings. The results of retrieved studies confirm the shortage of knowledge in this important area and confirm that most of the existing studies have problems with exposure misclassification that may have biased the summary estimates towards the null. Health impact/risk assessors should consider these limitations and future well-designed pregnancy cohort studies are needed to aid understanding of these important issues.

## Author Contributions

Evridiki Patelarou conceived the study, designed the study, performed the search, reviewed the papers and drafted the manuscript. Frank J. Kelly participated in the study design, articles’ review and helped to draft the manuscript. All authors read and approved the final manuscript.
